# Man’s Seven Ages

**Published:** 1892-01

**Authors:** 


### MAN'S SEVEN AGES.

The *Minneapolis Journal* is responsible for the following travesty of the immortal bard:

"Mrs. Jones has a baby."

"The deuce you say."

"Fact."

"Girl or boy?"

"Boy."

"Let's hunt up old Jonesey and make him set 'em up."

So is ushered into the world Baby Jones, very red and hungry, and very much troubled with insomnia. The former grows on him, and, on wearing off the latter, Papa Jones loses his hair and several pounds of flesh, and Mamma Jones loses some of her good looks.

"Pay!" says Papa Jones, in astonishment. "Do babies pay? Well, I should say they did."

"Pay?" says Mamma Jones; "why, the whole world could not buy him."

And so Baby Jones becomes Willie Jones, and with his little primer and immaculate tie marches proudly to school. Thereupon the boys "christen" his new shoes by spitting on them, and they soil his white tie rolling him over in the sand. And Willie Jones cries and teacher comforts him by letting him sit on the platform and by calling his tormentors "bad boys." And they grin and look ashamed.

But Father Time keeps his schythe a-swing, and lo! "Billy" Jones is in the high school. Billy the Kid he is called now, and he nearly breaks his mother's heart one day because she sees him smoking a cigarette and evidently enjoying it. Billy Jones is also inclined to partake of the fruit of his neighbor's pear tree, said fruit being obtained after dark.

Will Jones is a different boy a few years after his graduation from the high school. Life has become a question of neckties and fits on clothes. He ushers strangers into seats at the Church of the Dan and

Beersheba Pilgrims. He leads the german, and one day Deacon Potts is pained to see him coming out of a bucket shop, where he has taken a flyer on wheat.

William Jones, aged forty, is the cashier of the Hightop National Bank. Mr. Jones is known as one of our best and brainiest business men. Mrs. Jones speaks of him as Mr. Jones, or William, and Deacon Potts takes his advice on the investment of a few hundred dollars he has laid by. They talk of running him for the Legislature, and the Evening Squash has boomed him for Mayor. The little ones call him papa, and run to meet him at night when he comes home.

At sixty.

"You know old Bill Jones? He was telling me the other day how he used to play ball where the Post Office now stands. He's a jolly old fellow, I tell you. Told about helping to pitch a teacher out of the window when he was a boy, about forty or fifty years ago."

Billy is a jolly old boy. Kept the company in a roar at his daughter's wedding with his stories. They say Bill has laid by quite a little pile in his day. Smooth old boy is Bill. He has accumulated quite a stock of experience, at any rate, and is always ready with a word of counsel, if you ask his advice.

Seventy-five years old to-day is "Old Bill Jones," or "Old Billy Jones," as his younger friends love to call him. There is no term of reproach in familiarity unless it is used by the thoughtless or inconsiderate. His only friends have dropped by the wayside, one by one, and old Bill Jones is the last leaf on the tree. It is well ripened by time and frost, and the first breath of winter will detach him gently, and he will fall to his parent earth in sweet peace. "Old Bill Jones!" He has done his work well, and he is ready to go. He wonders if he will meet the old boys again and talk over the old days. His mind is much on his youth. He loves to recall the old associations. The old voices are in his ears. He smiles as he sees the children play.

"Hello, Colonel, whose grave is this you are filling?"

"William Jones, sir."

"What? Old Billy Jones? Well, well. So he has gone. But he lived to a good old age. Let's see, 'seventy-eight years, three months,' the stone says, don't it? That's doing pretty well in these times. Jones was a good old fellow, though. I remember hearing my father tell how Jones let him have $5,000 once to tide him over a crisis and he never took a bit of security. He and father were great friends once. How long ago? Oh, that must have been twenty or twenty-five years back. Father's been dead eighteen years. Well, good luck to him, wherever he is. Good day."

